# Seafood and polyunsaturated fatty acid intake and age-related hearing loss: a cross-sectional study conducted in Japan

**DOI:** 10.1016/j.jnha.2026.100971

**Published:** 2026-09-04

**Authors:** Jun Suzuki, Hiroki Tozuka, Hiyori Takahashi, Ikuko N. Motoike, Miyuki Sakurai, Yuta Kobayashi, Gosuke Watarai, Mana Kogure, Tetsuaki Kawase, Yohei Honkura, Ryoukichi Ikeda, Kengo Kinoshita, Naoki Nakaya, Taku Obara, Atsushi Hozawa, Shinichi Kuriyama, Nobuo Fuse, Masayuki Yamamoto, Yukio Katori

**Affiliations:** a Department of Otolaryngology, Head and Neck Surgery, Tohoku University Graduate School of Medicine, 1-1 Seiryo-machi, Aoba-ku, Sendai, Miyagi 980-8574, Japan; b Tohoku Medical Megabank Organization, Tohoku University, Sendai, Miyagi, Japan; c Department of Otolaryngology-Head and Neck Surgery, JCHO Sendai Hospital, Sendai, Miyagi, Japan; d Department of Preventive Medicine and Epidemiology, Tohoku Medical Megabank Organization, Tohoku University, Sendai, Miyagi, Japan; e Laboratory of Rehabilitative Auditory Science, Tohoku University Graduate School of Biomedical Engineering, Sendai, Miyagi, Japan; f Department of Otolaryngology, Head and Neck Surgery, Iwate Medical University School of Medicine, Yahaba, Iwate, Japan; g Advanced Research Center for Innovations in Next-Generation Medicine, Tohoku University, Sendai, Miyagi, Japan; h Department of Applied Information Sciences, Graduate School of Information Sciences, Tohoku University, Sendai, Miyagi, Japan; i International Research Institute of Disaster Science, Disaster Medical Science Division, Disaster-related Public Health, Tohoku University, Sendai, Miyagi, Japan; j Department of Integrative Genomics, Tohoku University, Sendai, Miyagi, Japan

**Keywords:** Age-related hearing loss, Seafood, Fatty acid, Dietary intake, Japanese

## Abstract

**Background:**

Age-related hearing loss (ARHL) is a common condition associated with dementia, social isolation, and reduced quality of life. N-3 polyunsaturated fatty acids (N-3 PUFAs), mainly derived from seafood, have been hypothesized to protect against ARHL through anti-inflammatory and antioxidant effects. However, evidence remains inconsistent. This cross-sectional study examined the association of seafood and dietary fatty acid intakes with ARHL in a Japanese population with high seafood consumption.

**Methods:**

A total of 13,908 adults aged 50–79 years from the Tohoku Medical Megabank Project Cohort Study were included. Hearing thresholds were measured by pure-tone audiometry at 500, 1,000, 2,000, and 4,000 Hz. Dietary intake was assessed using a validated food frequency questionnaire. Seafood, N-3 PUFA, and saturated fatty acid (SFA) intakes were categorized into quartiles. Multivariable linear regression was used to estimate adjusted mean differences in hearing thresholds.

**Results:**

In men, higher SFA intake was significantly associated with lower hearing thresholds at 1,000 Hz (Q4 vs. Q1: β = −2.23 dB HL, 95% CI: −3.437 to −1.023) and for pure-tone average (β = −1.704 dB HL; 95% CI, −2.872 to −0.536). Seafood and N-3 PUFA intakes were not significantly associated with hearing thresholds. In women, no significant associations were observed.

**Conclusion:**

In this population with high seafood consumption, seafood and N-3 PUFA intakes were not associated with hearing thresholds. Low SFA intake may be associated with higher hearing thresholds in Japanese men, suggesting that the relationship between dietary fatty acids and ARHL may be complex and may differ by sex.

## 1 Introduction

Age-related hearing loss (ARHL) is a common condition in older adults and is associated with impaired communication, social isolation, depression, and dementia [[1], [2], [3], [4]]. Therefore, identifying modifiable factors for ARHL is important.

Seafood, including fish and shellfish, is rich in N-3 polyunsaturated fatty acids (PUFAs), such as docosahexaenoic acid (DHA, 22:6 N-3) and eicosapentaenoic acid (EPA, 20:5 N-3), which have anti-inflammatory and antioxidant properties [5,6]. Considering the substantial evidence of protective effects of N-3 PUFAs on the central nervous and cardiovascular systems [7,8], N-3 PUFAs or seafood intakes may help prevent hearing loss. However, evidence remains inconsistent: N-3 PUFA supplementation and fish intake have been associated with both protective [[9], [10], [11], [12], [13]] and adverse effects on hearing loss [14,15].

The database of Tohoku Medical Megabank (TMM) Project includes detailed dietary questionnaire data and pure-tone audiometry results [[16], [17], [18]]. Because Japanese people consume more seafood and N-3 PUFAs than many other populations [6,19], this population is suitable for evaluating whether further increases in seafood or N-3 PUFA intake are associated with hearing preservation. Therefore, we aimed to examine the associations of seafood, N-3 PUFA, and saturated fatty acid (SFA) intakes with hearing thresholds in a large Japanese population.

## 2 Materials and methods

### 2.1 Study design and population

For this cross-sectional study, we used data from two cohort studies conducted by the Tohoku University Tohoku Medical Megabank Organization (ToMMo): the TMM Community-Based Cohort Study and the TMM Birth and Three-Generation Cohort Study [16,20]. This study was approved by the Ethical Research Committee of Tohoku University Graduate School of Medicine (2024-1-929), and written informed consent was obtained from all participants.

Among 25,009 participants aged ≥20 years who underwent pure-tone audiometry between July 2021 and January 2024, we excluded those with a history of chronic otitis media or missing information on otitis media history to minimize the influence of conductive hearing loss. In addition, we excluded participants aged < 50 years or ≥ 80 years because the effects of aging were expected to be limited in younger participants and the number of older participants was small. Participants with missing PUFA intake data or covariates for linear regression were also excluded. Finally, 13,908 participants were included (Supplementary Figure S1).

### 2.2 Audiometric assessments

The air-conduction thresholds were measured at 500, 1,000, 2,000, and 4,000 Hz using a pure-tone audiometer (AA-H1; RION, Tokyo, Japan) in a soundproof booth (AT-66; RION, Tokyo, Japan). The pure-tone average (PTA) was calculated as the mean threshold across these four frequencies for each ear, and the better-hearing ear was used for analysis. For descriptive analyses, hearing loss was defined as PTA > 25 decibels hearing level (dB HL), consistent with previous studies [18,21].

### 2.3 Dietary assessment and covariates

Dietary intake was assessed using the validated TMM food frequency questionnaire, which includes 138 food and beverage items [22]. Seafood intake was calculated from the intake volume of relevant food items. Nutrient intakes, including SFAs, monounsaturated fatty acids (MUFAs), PUFAs, and N-3 and n-6 PUFAs, were energy-adjusted using the residual method separately for men and women [[23], [24], [25]]. Blood DHA, EPA, and arachidonic acid levels were also used to assess the validity of the estimated seafood intake (Supplemental Materials and Methods).

Potential confounders included age, body mass index (BMI), family history of hearing loss, noisy occupation, education, smoking status, drinking status, diabetes mellitus (DM), hypertension (HT), dyslipidemia (DL), number of teeth, cohort type, daily energy intake, high-density lipoprotein cholesterol (HDL-C), low-density lipoprotein cholesterol (LDL-C), triglycerides (TG), and relevant dietary factors. DM, HT, and DL were defined using treatment history and measured clinical values. Noisy occupations were defined based on occupational categories previously reported to involve noise exposure [17]. Detailed definitions are provided in Supplemental Materials and Methods.

### 2.4 Statistical analysis

For comparison of background characteristics, continuous variables were analyzed using the Mann–Whitney U test, and categorical variables were analyzed using the two-tailed χ^2^ test. Statistical significance was set at P < 0.05, based on two-tailed tests. Data are presented as medians (with interquartile ranges [IQR]) for continuous variables and as numbers (%) for categorical variables.

To investigate the association between seafood intake and blood PUFA levels, seafood intake was classified into four categories based on quartiles of intake (Q1–Q4). Linear trend tests were performed by assigning scores from 1 to 4 to the quartiles and entering these scores into regression models.

Linear regression analysis was performed using hearing thresholds (dB HL) as the dependent variable, and β coefficients with 95% confidence intervals were estimated. In multivariable models examining seafood and N-3 PUFA intakes, covariates included age, body mass index, family history of hearing loss, noisy occupation, educational status, smoking status, drinking status, DM, HT, number of teeth, cohort type, energy intake, HDL-C, LDL-C, TG, MUFA intake, and SFA intake. In the models for SFA intake, seafood intake was included instead of SFA intake. Multicollinearity was assessed using variance inflation factors, and all values were <2. To account for multiple comparisons, P values were further adjusted using the Benjamini–Hochberg false discovery rate (FDR) method, with FDR-adjusted P values <0.10 considered statistically significant in multivariable analyses. All statistical analyses were performed using R version 4.2.1 (R Core Team, Vienna, Austria).

## 3 Results

### 3.1 Demographic characteristics of the participants

This study included 13,908 participants, comprising 4,957 men and 8,951 women. The median age was 65.0 [62.0–69.0] years for men and 62.0 [57.0–66.0] years for women. The PTA of the better-hearing ear was 26.2 [20.0–36.2] dB HL for men and 21.2 [15.0–28.8] dB HL for women.

Estimated seafood intake was positively associated with blood DHA and EPA levels in both men and women, supporting the validity of the dietary estimates (Supplementary Table S1).

In univariate analyses, participants with hearing loss were generally older, had a lower frequency of noisy occupations, a higher prevalence of cardiovascular disease, a lower proportion of participants from the TMM Birth and Three-Generation Cohort Study, a lower frequency of having household members, lower educational status, fewer teeth, higher energy intake per day, higher N-3:n-6 ratio, lower SFA intake, lower total cholesterol (TC) level, lower HDL-C level, and lower LDL-C level than those without hearing loss in both men and women (Table 1).

**Table 1:** Characteristics of participants according to hearing loss status by sex.

	Men					Women				
	Normal		Hearing loss		p-value	Normal		Hearing loss		p-value
Number	2062		2895			5558		3393		
PTA (dB HL)	17.5 [15.0, 21.2]		33.8 [28.8, 41.2]		<0.001	16.2 [12.5, 20.0]		31.2 [27.5, 37.5]		<0.001
Age (years)	64.0 [59.0, 67.0]		67.0 [64.0, 70.0]		<0.001	60.0 [56.0, 64.0]		65.0 [61.0, 69.0]		<0.001
50−59	523	25.4%	246	8.5%	<0.001	2523	45.4%	547	16.1%	<0.001
60−69	1262	61.2%	1747	60.3%		2716	48.9%	2057	60.6%	
70−79	277	13.4%	902	31.2%		319	5.7%	789	23.3%	
BMI (kg/m²)	23.6 [21.9, 25.5]		23.7 [22.0, 25.6]		0.236	22.4 [20.3, 24.7]		22.6 [20.6, 25.1]		<0.001
Family history of hearing loss, n (%)	64	3.1%	76	2.6%	0.36	236	4.2%	148	4.4%	0.833
Noise risk occupation, n (%)	158	7.7%	160	5.5%	0.003	216	3.9%	102	3.0%	0.034
History of cardiovascular disease, n (%)	353	17.1%	584	20.2%	0.008	402	7.2%	313	9.2%	<0.001
Type of cohort, n (%)										
Community-Based Cohort	1678	81.4%	2585	89.3%	<0.001	4484	80.7%	2981	87.9%	<0.001
Birth and Three-Generation Cohort	384	18.6%	310	10.7%		1074	19.3%	412	12.1%	
Living with spouse, n (%)	1857	90.1%	2570	88.9%	0.207	4240	76.4%	2405	70.9%	<0.001
Living with household members, n (%)	1937	93.9%	2664	92.0%	0.012	4882	87.8%	2856	84.2%	<0.001
Educational status, n (%)					<0.001					0.001
High (college and above)	703	34.1%	647	22.3%		520	9.4%	194	5.7%	<0.001
Middle (high school)	1236	59.9%	1859	64.2%		4774	86.0%	2915	85.9%	
Low (junior high school and below)	116	5.6%	383	13.2%		256	4.6%	281	8.3%	
Others	7	0.3%	6	0.2%		8	0.1%	3	0.1%	
Smoking status, n (%)					0.332					<0.001
Never (< = 100)	576	27.9%	757	26.1%		4772	85.9%	3098	91.3%	
Past	1258	61.0%	1823	63.0%		601	10.8%	235	6.9%	
Current(> = 100)	228	11.1%	315	10.9%		185	3.3%	60	1.8%	
Drinking status, n (%)					0.983					<0.001
Never	426	20.7%	600	20.7%		3211	57.8%	2166	63.9%	
Past / Current	1636	79.3%	2295	79.3%		2347	42.2%	1226	36.1%	
DM, n (%)	321	15.6%	498	17.2%	0.137	363	6.5%	363	10.7%	<0.001
HT, n (%)	1495	72.5%	2131	73.6%	0.404	3015	54.2%	2191	64.6%	<0.001
DL, n (%)	353	17.1%	418	14.4%	0.012	1158	20.8%	735	21.7%	0.352
Number of teeth, n (%)					<0.001					<0.001
>= 20	1613	78.2%	1957	67.6%		4579	82.4%	2431	71.6%	
10 to 19	261	12.7%	551	19.0%		692	12.5%	649	19.1%	
< 9	188	9.1%	387	13.4%		287	5.2%	313	9.2%	
Energy intake per day (kcal)	1960.7 [1570.1, 2396.4]		1999.9 [1593.9, 2447.9]		0.018	1786.5 [1432.8, 2216.3]		1812.9 [1451.7, 2292.1]		0.013
Seafood intake (g)	56.8 [34.3, 87.4]		59.8 [34.5, 90.2]		0.104	59.8 [38.2, 89.4]		63.3 [39.1, 98.3]		<0.001
n-3 PUFA intake (g)	2.3 [1.8, 2.8]		2.3 [1.9, 2.9]		0.039	2.5 [2.1, 3.0]		2.6 [2.1, 3.0]		0.068
n-6 PUFA intake (g)	11.3 [9.4, 13.2]		11.2 [9.4, 13.1]		0.846	12.2 [10.6, 14.0]		12.2 [10.5, 14.0]		0.303
n-3:n-6 ratio	0.201 [0.172, 0.232]		0.204 [0.174, 0.239]		0.014	0.203 [0.176, 0.234]		0.206 [0.179, 0.240]		0.001
PUFA intake (g)	13.7 [11.4, 16.0]		13.7 [11.5, 16.0]		0.722	14.9 [13.0, 17.0]		14.9 [12.8, 17.0]		0.689
MUFA intake (g)	24.4 [19.9, 28.8]		23.9 [19.9, 28.5]		0.182	27.3 [23.7, 31.1]		27.1 [23.3, 31.0]		0.175
SFA intake (g)	20.5 [16.8, 24.6]		20.1 [16.3, 24.0]		0.003	23.1 [19.7, 26.6]		22.9 [19.4, 26.3]		0.023
TC (mg/dL)	194.0 [172.0, 217.0]		188.0 [167.0, 209.0]		<0.001	215.0 [193.0, 237.0]		210.0 [188.0, 232.0]		<0.001
HDL-C (mg/dL)	57.0 [47.0, 68.0]		55.0 [47.0, 67.0]		0.029	68.0 [58.0, 81.0]		66.0 [56.0, 78.0]		<0.001
LDL-C (mg/dL)	113.0 [92.2, 133.0]		108.0 [89.0, 127.0]		<0.001	123.0 [104.0, 144.0]		120.0 [100.0, 140.0]		<0.001
TG (mg/dL)	115.0 [80.0, 169.0]		113.0 [79.0, 166.0]		0.218	101.0 [73.0, 144.0]		106.0 [76.0, 148.0]		<0.001

PTA, pure-tone average; dB HL, decibel hearing level; BMI, body mass index; DM, diabetes mellitus; HT, hypertension; DL, dyslipidemia; PUFA, polyunsaturated fatty acid; MUFA, monounsaturated fatty acid; SFA, saturated fatty acid; TC, total cholesterol; HDL-C, high-density lipoprotein cholesterol; LDL-C, low-density lipoprotein cholesterol; TG, triglycerides.

### 3.2 Associations of seafood, N-3 PUFA, and SFA intakes with hearing thresholds

In men, multivariable linear regression showed no significant association between seafood or N-3 PUFA intake and hearing thresholds at any frequency or for PTA after FDR correction (Table 2). However, SFA intake was inversely associated with hearing thresholds at 1,000 Hz (β = −2.23 dB HL; 95% CI, −3.437 to −1.023; FDR-adjusted P value = 0.006) and for PTA (β = −1.704 dB HL; 95% CI, −2.872 to −0.536; FDR-adjusted P value = 0.089), with the Q4 group showing lower thresholds than the Q1 group.

**Table 2:** Multivariable-adjusted β coefficients for hearing thresholds (dB HL) according to quartiles of seafood, N-3 PUFA, and SFA intake in men.

Men, n = 4957	Seafood						n-3 PUFA						SFA				
PTA	β coefficient	95% CI: lower	95% CI: upper	p-value	FDR-adjusted P value	PTA	β coefficient	95% CI: lower	95% CI: upper	p-value	FDR-adjusted P value	PTA	β coefficient	95% CI: lower	95% CI: upper	p-value	FDR-adjusted P value
Q1 (< 34.3 g)	Reference	—	—	—	—	Q1(< 1.8 g)	Reference	—	—	—	—	Q1 (< 16.5 g)	Reference	—	—	—	—
Q2 (34.3–58.4 g)	−0.86	−1.744	−0.025	0.057	0.298	Q2 (1.8–2.3 g)	−0.346	−1.245	−0.554	0.451	0.677	Q2 (16.5–20.3 g)	0.012	−0.912	−0.937	0.979	0.983
Q3 (58.4–88.9 g)	−0.226	−1.116	−0.665	0.619	0.807	Q3 (2.3–2.8 g)	−0.179	−1.11	−0.751	0.705	0.823	Q3 (20.3–24.2 g)	−1.059	−2.083	−0.034	0.043	0.298
Q4 (> = 88.9 g)	−0.547	−1.429	−0.335	0.224	0.541	Q4 (> = 2.8 g)	−0.794	−1.75	−0.162	0.104	0.436	Q4 (> = 24.2 g)	−1.704	−2.872	−0.536	0.004	0.089
500 Hz	β coefficient	95% CI: lower	95% CI: upper	p-value	FDR-adjusted P value	500 Hz	β coefficient	95% CI: lower	95% CI: upper	p-value	FDR-adjusted P value	500 Hz	β coefficient	95% CI: lower	95% CI: upper	p-value	FDR-adjusted P value
Q1 (< 34.3 g)	Reference	—	—	—	—	Q1(< 1.8 g)	Reference	—	—	—	—	Q1 (< 16.5 g)	Reference	—	—	—	—
Q2 (34.3–58.4 g)	−0.381	−1.242	−0.48	0.386	0.723	Q2 (1.8–2.3 g)	−0.497	−1.371	−0.378	0.266	0.613	Q2 (16.5–20.3 g)	0.223	−0.677	−1.123	0.627	0.852
Q3 (58.4–88.9 g)	0.149	−0.718	−1.016	0.736	0.909	Q3 (2.3–2.8 g)	0.487	−0.418	−1.392	0.292	0.613	Q3 (20.3–24.2 g)	−0.571	−1.568	−0.427	0.262	0.613
Q4 (> = 88.9 g)	−0.199	−1.058	−0.66	0.649	0.852	Q4 (> = 2.8 g)	−0.561	−1.491	−0.37	0.238	0.613	Q4 (> = 24.2 g)	−1.228	−2.364	−0.091	0.034	0.613
1000 Hz	β coefficient	95% CI: lower	95% CI: upper	p-value	FDR-adjusted P value	1000 Hz	β coefficient	95% CI: lower	95% CI: upper	p-value	FDR-adjusted P value	1000 Hz	β coefficient	95% CI: lower	95% CI: upper	p-value	FDR-adjusted P value
Q1 (< 34.3 g)	Reference	—	—	—	—	Q1(< 1.8 g)	Reference	—	—	—	—	Q1 (< 16.5 g)	Reference	—	—	—	—
Q2 (34.3–58.4 g)	−0.673	−1.587	−0.241	0.149	0.783	Q2 (1.8–2.3 g)	−0.205	−1.134	−0.724	0.665	0.977	Q2 (16.5–20.3 g)	−0.715	−1.671	−0.241	0.143	0.783
Q3 (58.4–88.9 g)	0.123	−0.798	−1.043	0.794	0.977	Q3 (2.3–2.8 g)	0.45	−0.512	−1.412	0.359	0.977	Q3 (20.3–24.2 g)	−1.295	−2.354	−0.235	0.017	0.174
Q4 (> = 88.9 g)	−0.11	−1.022	−0.803	0.814	0.977	Q4 (> = 2.8 g)	−0.378	−1.367	−0.611	0.453	0.977	Q4 (> = 24.2 g)	−2.23	−3.437	−1.023	<0.001	0.006
2000 Hz	β coefficient	95% CI: lower	95% CI: upper	p-value	FDR-adjusted P value	2000 Hz	β coefficient	95% CI: lower	95% CI: upper	p-value	FDR-adjusted P value	2000 Hz	β coefficient	95% CI: lower	95% CI: upper	p-value	FDR-adjusted P value
Q1 (< 34.3 g)	Reference	—	—	—	—	Q1(< 1.8 g)	Reference	—	—	—	—	Q1 (< 16.5 g)	Reference	—	—	—	—
Q2 (34.3–58.4 g)	−1.118	−2.228	−0.007	0.048	0.255	Q2 (1.8–2.3 g)	−0.223	−1.352	−0.906	0.699	0.982	Q2 (16.5–20.3 g)	0.056	−1.105	−1.217	0.924	0.982
Q3 (58.4–88.9 g)	−0.292	−1.41	−0.827	0.609	0.982	Q3 (2.3–2.8 g)	−0.311	−1.479	−0.857	0.602	0.982	Q3 (20.3–24.2 g)	−1.098	−2.384	−0.189	0.094	0.397
Q4 (> = 88.9 g)	−0.565	−1.673	−0.543	0.317	0.833	Q4 (> = 2.8 g)	−0.888	−2.089	−0.313	0.147	0.515	Q4 (> = 24.2 g)	−1.627	−3.093	−0.161	0.03	0.255
4000 Hz	β coefficient	95% CI: lower	95% CI: upper	p-value	FDR-adjusted P value	4000 Hz	β coefficient	95% CI: lower	95% CI: upper	p-value	FDR-adjusted P value	4000 Hz	β coefficient	95% CI: lower	95% CI: upper	p-value	FDR-adjusted P value
Q1 (< 34.3 g)	Reference	—	—	—	—	Q1(< 1.8 g)	Reference	—	—	—	—	Q1 (< 16.5 g)	Reference	—	—	—	—
Q2 (34.3–58.4 g)	−0.929	−2.301	−0.442	0.184	0.268	Q2 (1.8–2.3 g)	−0.512	−1.906	−0.882	0.471	0.556	Q2 (16.5–20.3 g)	0.476	−0.958	−1.91	0.515	0.556
Q3 (58.4–88.9 g)	−0.484	−1.865	−0.897	0.492	0.556	Q3 (2.3–2.8 g)	−1.088	−2.531	−0.355	0.139	0.254	Q3 (20.3–24.2 g)	−1.181	−2.77	−0.407	0.145	0.254
Q4 (> = 88.9 g)	−1.125	−2.493	−0.244	0.107	0.254	Q4 (> = 2.8 g)	−1.553	−3.036	−0.071	0.04	0.254	Q4 (> = 24.2 g)	−1.79	−3.601	−0.02	0.053	0.254

PTA, pure-tone average; Q1, lowest quartile; Q2, second quartile; Q3, third quartile; Q4, highest quartile; CI, confidence interval; PUFA, polyunsaturated fatty acid; SFA, saturated fatty acid; dB HL, decibel hearing level.

In women, no significant associations were observed between seafood, N-3 PUFA, or SFA intake and hearing thresholds at any frequency or for PTA after FDR correction (Table 3).

**Table 3:** Multivariable-adjusted β coefficients for hearing thresholds (dB HL) according to quartiles of seafood, N-3 PUFA, and SFA intake in women.

Women, n = 8951	Seafood						n-3 PUFA						SFA				
PTA	β coefficient	95% CI: lower	95% CI: upper	p-value	FDR-adjusted P value	PTA	β coefficient	95% CI: lower	95% CI: upper	p-value	FDR-adjusted P value	PTA	β coefficient	95% CI: lower	95% CI: upper	p-value	FDR-adjusted P value
Q1 (< 38.6 g)	Reference	—	—	—	—	Q1(< 2.1 g)	Reference	—	—	—	—	Q1 (< 19.6 g)	Reference	—	—	—	—
Q2 (38.6–61.2 g)	−0.284	−0.847	0.279	0.322	0.836	Q2 (2.1–2.5 g)	−0.251	−0.832	0.331	0.398	0.836	Q2 (19.6–23.0 g)	−0.408	−0.987	0.171	0.167	0.836
Q3 (61.2–92.9 g)	−0.169	−0.731	0.394	0.557	0.964	Q3 (2.5–3.0 g)	0.132	−0.459	0.724	0.661	0.964	Q3 (23.0–26.5 g)	0.022	−0.582	0.625	0.944	0.964
Q4 (> = 92.9 g)	0.557	−0.004	1.119	0.052	0.836	Q4 (> = 3.0 g)	−0.428	−1.026	0.169	0.16	0.836	Q4 (> = 26.5 g)	−0.28	−0.915	0.355	0.387	0.836
500 Hz	β coefficient	95% CI: lower	95% CI: upper	p-value	FDR-adjusted P value	500 Hz	β coefficient	95% CI: lower	95% CI: upper	p-value	FDR-adjusted P value	500 Hz	β coefficient	95% CI: lower	95% CI: upper	p-value	FDR-adjusted P value
Q1 (< 38.6 g)	Reference	—	—	—	—	Q1(< 2.1 g)	Reference	—	—	—	—	Q1 (< 19.6 g)	Reference	—	—	—	—
Q2 (38.6–61.2 g)	0.214	−0.368	0.795	0.472	0.781	Q2 (2.1–2.5 g)	−0.143	−0.744	0.459	0.642	0.793	Q2 (19.6–23.0 g)	−0.146	−0.744	0.453	0.633	0.793
Q3 (61.2–92.9 g)	0.146	−0.436	0.727	0.624	0.793	Q3 (2.5–3.0 g)	0.219	−0.393	0.831	0.484	0.781	Q3 (23.0–26.5 g)	0.259	−0.365	0.883	0.415	0.781
Q4 (> = 92.9 g)	0.711	0.13	1.292	0.016	0.213	Q4 (> = 3.0 g)	−0.188	−0.806	0.429	0.55	0.793	Q4 (> = 26.5 g)	0.009	−0.647	0.665	0.979	0.979
1000 Hz	β coefficient	95% CI: lower	95% CI: upper	p-value	FDR-adjusted P value	1000 Hz	β coefficient	95% CI: lower	95% CI: upper	p-value	FDR-adjusted P value	1000 Hz	β coefficient	95% CI: lower	95% CI: upper	p-value	FDR-adjusted P value
Q1 (< 38.6 g)	Reference	—	—	—	—	Q1(< 2.1 g)	Reference	—	—	—	—	Q1 (< 19.6 g)	Reference	—	—	—	—
Q2 (38.6–61.2 g)	−0.263	−0.874	0.347	0.398	0.76	Q2 (2.1–2.5 g)	−0.306	−0.938	0.325	0.341	0.717	Q2 (19.6–23.0 g)	−0.574	−1.203	0.055	0.074	0.475
Q3 (61.2–92.9 g)	−0.025	−0.635	0.585	0.936	0.983	Q3 (2.5–3.0 g)	−0.05	−0.693	0.325	0.341	0.717	Q3 (23.0–26.5 g)	−0.347	−1.002	0.308	0.299	0.698
Q4 (> = 92.9 g)	0.601	−0.009	−1.21	0.053	0.475	Q4 (> = 3.0 g)	−0.508	−1.156	0.14	0.125	0.475	Q4 (> = 26.5 g)	−0.496	−1.185	0.193	0.158	0.475
2000 Hz	β coefficient	95% CI: lower	95% CI: upper	p-value	FDR-adjusted P value	2000 Hz	β coefficient	95% CI: lower	95% CI: upper	p-value	FDR-adjusted P value	2000 Hz	β coefficient	95% CI: lower	95% CI: upper	p-value	FDR-adjusted P value
Q1 (< 38.6 g)	Reference	—	—	—	—	Q1(< 2.1 g)	Reference	—	—	—	—	Q1 (< 19.6 g)	Reference	—	—	—	—
Q2 (38.6–61.2 g)	−0.452	−1.132	0.228	0.193	0.914	Q2 (2.1–2.5 g)	−0.349	−1.052	0.354	0.331	0.914	Q2 (19.6–23.0 g)	−0.367	−1.067	0.333	0.304	0.914
Q3 (61.2–92.9 g)	−0.239	−0.918	0.441	0.491	0.914	Q3 (2.5–3.0 g)	0.136	−0.579	0.852	0.709	0.914	Q3 (23.0–26.5 g)	0.273	−0.457	1.002	0.464	0.914
Q4 (> = 92.9 g)	0.596	−0.083	1.275	0.085	0.914	Q4 (> = 3.0 g)	−0.364	−1.086	0.358	0.323	0.914	Q4 (> = 26.5 g)	−0.121	−0.888	0.647	0.758	0.914
4000 Hz	β coefficient	95% CI: lower	95% CI: upper	p-value	FDR-adjusted P value	4000 Hz	β coefficient	95% CI: lower	95% CI: upper	p-value	FDR-adjusted P value	4000 Hz	β coefficient	95% CI: lower	95% CI: upper	p-value	FDR-adjusted P value
Q1 (< 38.6 g)	Reference	—	—	—	—	Q1(< 2.1 g)	Reference	—	—	—	—	Q1 (< 19.6 g)	Reference	—	—	—	—
Q2 (38.6–61.2 g)	−0.601	−1.377	0.175	0.129	0.994	Q2 (2.1–2.5 g)	−0.264	−1.066	0.538	0.519	0.994	Q2 (19.6–23.0 g)	−0.679	−1.478	0.12	0.096	0.994
Q3 (61.2–92.9 g)	−0.45	−1.225	0.325	0.255	0.994	Q3 (2.5–3.0 g)	0.073	−0.744	0.889	0.861	0.994	Q3 (23.0–26.5 g)	−0.026	−0.859	0.806	0.95	0.994
Q4 (> = 92.9 g)	0.325	−0.45	1.099	0.411	0.994	Q4 (> = 3.0 g)	−0.455	−1.279	0.369	0.279	0.994	Q4 (> = 26.5 g)	−0.4	−1.276	0.475	0.37	0.994

PTA, pure-tone average; Q1, lowest quartile; Q2, second quartile; Q3, third quartile; Q4, highest quartile; CI, confidence interval; PUFA, polyunsaturated fatty acid; SFA, saturated fatty acid; dB HL, decibel hearing level.

Because age had a strong influence in these analyses, we stratified both men and women into two age groups—50–64 years (Supplementary Table S2) and 65–79 years (Supplementary Table S3)—and performed the same analyses. As in the analysis of the overall population, no significant associations were observed among women; however, SFA intake was inversely associated with hearing thresholds at 1,000 Hz (β = −2.564 dB HL; 95% CI, −4.262 to −0.866; FDR-adjusted P value = 0.065) among men aged 65–79 years, with the Q4 group showing lower thresholds than the Q1 group (Supplementary Tables S4 and S5).

## 4 Discussion

In this large Japanese population with relatively high seafood intake, seafood and N-3 PUFA intakes were not significantly associated with hearing thresholds in either sex. In contrast, higher SFA intake was associated with slightly lower hearing thresholds at 1,000 Hz and for PTA in men.

Previous cohort studies, including the Blue Mountains Hearing Study, Nurses’ Health Study II, and UK Biobank studies, have reported that higher fish or N-3 PUFA intake, higher plasma N-3 PUFA levels, and replacing SFAs with PUFAs are associated with a lower risk of hearing loss [[11], [12], [13],^26^]. However, our findings suggest that, in a population that already consumes relatively large amounts of seafood, further increases in seafood or N-3 PUFA intake may not provide additional hearing protection. A previous comparative study indicated that Japanese people consume more fish than Caucasian Americans [27], supporting the possibility of a ceiling effect. Moreover, given reports of PUFA-related ototoxicity in animal studies [15,28,29] and potential cardiovascular risks of fish oil supplementation in the general population (40), the relationship between N-3 PUFAs and hearing may not be linear and may depend on individual health status.

The inverse association between SFA intake and hearing thresholds in men should be interpreted cautiously. Although excessive consumption of SFAs is associated with an increased risk of cardiovascular diseases [30], the men in the present study had relatively low SFA intake compared with U.S. dietary data: whereas the mean SFA intake among Americans in their 60 s was 30.8 g/day according to the U.S. Department of Agriculture “What We Eat in America” survey, even the highest quartile in men had an SFA intake of 24.2 g/day in this study. Therefore, our findings may reflect higher hearing thresholds among participants with relatively low SFA intake rather than a protective effect of high SFA intake. Because SFA-rich foods contain various nutrients and evidence on SFA intake and hearing loss remains limited, further studies are needed to clarify whether low SFA intake, specific food sources of SFAs, overall nutritional status, or dietary patterns are related to hearing loss.

This study has some limitations. Its cross-sectional design precludes causal inference. Residual confounding and chance findings cannot be excluded. Information on fish oil supplements and lipid-lowering medications was unavailable. In addition, as we did not perform otoscopic examinations and bone-conduction testing, conductive hearing loss may have been included. However, because the prevalence of middle ear disease and non-ARHL sensorineural hearing loss is relatively low, these conditions are unlikely to have substantially affected our evaluation of the association. Regarding noise exposure, an assessment based solely on industry sector may not have adequately identified participants exposed to occupational noise. Despite these limitations, this study is strengthened by its large cohort, audiometry-based hearing assessment, dietary and blood PUFA data, and inclusion of a Japanese population with relatively high seafood intake.

## 5 Conclusion

In this population with relatively high seafood intake, seafood and N-3 PUFA intakes were not associated with hearing thresholds, whereas low SFA intake may be associated with higher hearing thresholds in men. Further prospective studies are needed to clarify the role of dietary fatty acid intake in hearing preservation.

## Author contributions

Study design: JS, HT, and HT. Data analysis: HT, HT, JS, INM, MS, YK, GW, TK, YH, RI, AH, and NF. Cohort organization: MK, NN, TO, AH, and SK. Biobank organization: KK. The draft of the manuscript was written by JS. Supervision of research: MY and YK. All authors read and reviewed the final manuscript.

## Ethics statement

The present study was approved by the Ethical Research Committee of Tohoku University Graduate School of Medicine (2024-1-929), and written informed consent was obtained from all participants.

## Declaration of Generative AI and AI-assisted technologies in the writing process

All AI tools, including ChatGPT (OpenAI, San Francisco, USA), Grammarly (Grammarly Inc., San Francisco, USA), and DeepL (DeepL SE, Köln, Germany), were used solely to improve the clarity and readability of the English text. The authors take full responsibility for the content of this manuscript.

## Funding

This study was supported by JSPS KAKENHI (grant number: JP23KK0156), the Food and Health Research Fellowship Program of Honjo International Scholarship Foundation, and the Osake-no-Kagaku Foundation.

## Data Availability

The data used in this study are stored at Tohoku Medical Megabank Organization. The stored data will be shared upon reasonable request (contact us at dist@megabank.tohoku.ac.jp) after assembling the dataset and receiving approval from the Ethics Committee and the Materials and Information Distribution Review Committee of Tohoku Medical Megabank Organization.

## Declaration of competing interest

JS received research grants from the Osake-no-Kagaku Foundation and Honjo International Scholarship Foundation. The other authors have no conflicts of interest to declare.
