# Supplementary material for: Seafood and polyunsaturated fatty acid intake and age-related hearing loss: a cross-sectional study conducted in Japan
Source: J Nutr Health Aging. 2026 Sep 4;30(11):100971. doi: 10.1016/j.jnha.2026.100971 (PMC13571989; doi:10.1016/j.jnha.2026.100971)
Supplement: Supplementary file 1 [file mmc1.docx]

**Supplementary Materials and Methods**

1. Details of two cohort studies

This study used data from two cohort studies: the TMM Community-Based Cohort Study (TMM CommCohort Study) and the TMM Birth and Three-Generation Cohort Study (TMM BirThree Cohort Study) ^1, 2^. The TMM CommCohort Study included adult participants who underwent baseline assessments between July 2013 and March 2016 at specific health check-up sites. The TMM BirThree Cohort Study is a family-based cohort study that included pregnant women and their families who underwent the baseline assessments between July 2013 and March 2017. Basic information and biological samples were collected from the participants.

A total of 25,009 participants from the two aforementioned cohort studies, aged 20 years or older and residing in Miyagi Prefecture, Japan, underwent pure-tone audiometry at four frequencies (500, 1,000, 2,000, and 4,000 Hz) as part of tertiary assessments conducted between July 2021 and January 2024. After excluding participants as shown in Supplementary Figure 1, a total of 13,908 participants were included in the final analysis.

2. Quantification of plasma PUFA levels

ToMMo performed targeted metabolomics as a baseline assessment for some participants using the MxP Quant 500 Kit (Biocrates Life Sciences AG, Innsbruck, Austria) and various UHPLC-MS/MS systems ^3^. Of the 439 lipids measured, we evaluated the relationship between docosahexaenoic acid (DHA, 22:6 n-3), eicosapentaenoic acid (EPA, 20:5 n-3), and arachidonic acid (AA, C20:4 n-6) concentrations and seafood intake, as calculated using data from the TMM-FFQ administered during baseline assessments.

3. Assessment of participant characteristics and covariates

A detailed self-report questionnaire was administered to evaluate the participants’ characteristics. Body mass index was calculated by dividing weight (kg) by the square of height (m^2^). “Noisy occupations” were defined as those in the forestry, mining, construction, and manufacturing industries, as previously reported ^4^. Information on the history of cardiovascular disease and chronic otitis media, current medical conditions, and family history of hearing loss was assessed. Educational status was classified into four categories: university/graduate school; high school/technical school/junior college/technical college; elementary/junior high school; and others. Smoking status was classified into three categories: never smokers (smoked < 100 cigarettes in their lifetime), former smokers (smoked ≥ 100 cigarettes in their lifetime and currently not smoking), and current smokers (smoked ≥ 100 cigarettes in their lifetime and currently smoking). Drinking status was classified into two categories: never drinker and former/current drinker. Participants with hypertension were defined as those who reported being on treatment for hypertension, those with a systolic blood pressure of 140 mmHg or higher, or those with a diastolic blood pressure of 90 mmHg or higher. Participants with diabetes mellitus were defined as those who reported being on treatment for diabetes mellitus, those with a hemoglobin A1c level ≥ 6.5%, or those with a casual blood glucose level ≥ 200 mg/dL. Participants with dyslipidemia were defined as those who reported being on treatment for dyslipidemia. The number of teeth was determined through dental examination and divided into three groups: [1] 0–9, [2] 10–19, and [3] 20–28 teeth.

**References**

1) Kuriyama S, Yaegashi N, Nagami F, et al. The Tohoku Medical Megabank Project: Design and Mission. J Epidemiol. 2016;26(9):493-511.

2) Kuriyama S, Metoki H, Kikuya M, et al. Cohort Profile: Tohoku Medical Megabank Project Birth and Three-Generation Cohort Study (TMM BirThree Cohort Study): rationale, progress and perspective. Int J Epidemiol. 2020;49(1):18-9m.

3) Sato M, Hishinuma E, Matsukawa N, et al. Dietary habits and plasma lipid concentrations in a general Japanese population. Metabolomics. 2024;20(2):34.

4) Watarai G, Suzuki J, Motoike IN, et al. Relationship between age-related hearing loss and consumption of coffee and tea. Geriatr Gerontol Int. 2023;23(6):453-6.
