# Supplementary material for: Seafood and polyunsaturated fatty acid intake and age-related hearing loss: a cross-sectional study conducted in Japan
Source: J Nutr Health Aging. 2026 Sep 4;30(11):100971. doi: 10.1016/j.jnha.2026.100971 (PMC13571989; doi:10.1016/j.jnha.2026.100971)
Supplement: Supplementary file 2 [file mmc2.docx]

**Supplementary Table 1. Comparison of blood polyunsaturated fatty acid concentrations by seafood intake level**

Q1, lowest quartile; Q2, second quartile; Q3, third quartile; Q4, highest quartile; β, regression coefficient

| Men (n=1375) | Q1 (< 34.5 g) |  | Q2 (34.5 - 58.5 g) |  | Q3 (58.5 - 89.1 g) |  | Q4 ( >= 89.1 g) |  | p value | β | p for trend |
| --- | --- | --- | --- | --- | --- | --- | --- | --- | --- | --- | --- |
| Arachidonic acid (C20:4) [μM] | 7.4 [5.2, 9.7] |  | 8.1 [5.8, 10.5] |  | 7.5 [5.3, 10.0] |  | 8.1 [5.8, 10.5] |  | 0.774 | 0.0003 | 0.911 |
| Docosahexaenoic acid (C22:6) [μM] | 40.1 [24.5, 62.3] |  | 48.3 [28.9, 68.3] |  | 47.1 [28.4, 70.3] |  | 51.8 [29.7, 79.5] |  | <0.001 | 0.052 | <0.001 |
| Eicosapentaenoic acid (C20:5) [μM] | 4.3 [2.5, 6.8] |  | 5.5 [2.9, 7.9] |  | 5.2 [3.2, 8.5] |  | 6.1 [3.6, 9.9] |  | <0.001 | 0.009 | <0.001 |
|  |  |  |  |  |  |  |  |  |  |  |  |
| Women (n=2031) | Q1 (< 38.7 g) |  | Q2 (38.7 - 61.2 g) |  | Q3 (61.2 - 92.9 g) |  | Q4 ( >= 92.9 g) |  | p value | β | p for trend |
| Arachidonic acid (C20:4) [μM] | 8.4 [6.0, 11.2] |  | 8.4 [6.0, 11.2] |  | 8.6 [6.4, 11.0] |  | 8.7 [6.3, 11.5] |  | 0.999 | -0.001 | 0.661 |
| Docosahexaenoic acid (C22:6) [μM] | 42.8 [25.6, 64.7] |  | 47.3 [26.9, 69.8] |  | 52.8 [32.6, 74.1] |  | 56.1 [34.6, 78.2] |  | 0.004 | 0.036 | <0.001 |
| Eicosapentaenoic acid (C20:5) [μM] | 4.2 [2.4, 6.5] |  | 5.0 [3.0, 7.6] |  | 5.5 [3.6, 8.2] |  | 6.1 [4.0, 10.0] |  | <0.001 | 0.009 | <0.001 |

**Supplementary Table 2. Characteristics of participants according to hearing loss status by sex among those aged 50–64 years**

PTA, pure-tone average; dB HL, decibel hearing level; BMI, body mass index; DM, diabetes mellitus; HT, hypertension; DL, dyslipidemia; PUFA, polyunsaturated fatty acid; MUFA, monounsaturated fatty acid; SFA, saturated fatty acid; TC, total cholesterol; HDL-C, high-density lipoprotein cholesterol; LDL-C, low-density lipoprotein cholesterol; TG, triglyceride

| 50–64 years | Men | | | | | Women | | | | |
| --- | --- | --- | --- | --- | --- | --- | --- | --- | --- | --- |
|  | Normal |  | Hearing loss |  | p-value | Normal |  | Hearing loss |  | p-value |
| Number | 1185 |  | 925 |  |  | 4230 |  | 1525 |  |  |
| PTA (dB HL) | 17.5 [13.8, 21.2] |  | 31.2 [27.5, 37.5] |  | <0.001 | 16.2 [12.5, 20.0] |  | 30.0 [26.2, 35.0] |  | <0.001 |
| Age (years) | 60 [56, 62] |  | 62 [59, 63] |  | <0.001 | 58 [54, 61] |  | 61 [58, 63] |  | <0.001 |
| BMI (kg/m²) | 23.8 [22, 25.7] |  | 24.1 [22.4, 26.1] |  | 0.006 | 22.2 [20.2, 24.5] |  | 22.7 [20.4, 25] |  | <0.001 |
| Family history of hearing loss, n (%) | 44 | 3.7% | 37 | 4.0% | 0.821 | 204 | 4.8% | 76 | 5.0% | 0.856 |
| Noise risk occupation, n (%) | 134 | 11.3% | 89 | 9.6% | 0.238 | 195 | 4.6% | 62 | 4.1% | 0.418 |
| History of cardiovascular disease, n (%) | 180 | 15.2% | 161 | 17.4% | 0.189 | 258 | 6.1% | 97 | 6.4% | 0.763 |
| Type of cohort, n (%) |  |  |  |  |  |  |  |  |  |  |
| Community-Based Cohort | 923 | 77.9% | 763 | 82.5% | <0.001 | 3307 | 78.2% | 1243 | 81.5% | 0.007 |
| Birth and Three-Generation Cohort | 262 | 22.1% | 162 | 17.5% |  | 923 | 21.8% | 282 | 18.5% |  |
| Living with spouse, n (%) | 1063 | 89.7% | 803 | 86.8% | 0.054 | 3356 | 79.3% | 1203 | 78.9% | 0.736 |
| Living with household members, n (%) | 1104 | 93.2% | 848 | 91.7% | 0.228 | 3809 | 90.0% | 1363 | 89.4% | 0.488 |
| Educational status, n (%) |  |  |  |  | <0.001 |  |  |  |  | <0.001 |
| High (college and above) | 416 | 35.1% | 199 | 21.5% |  | 412 | 9.7% | 94 | 6.2% |  |
| Middle (high school) | 719 | 60.7% | 633 | 68.4% |  | 3668 | 86.7% | 1346 | 88.3% |  |
| Low (junior high school and below) | 47 | 4.0% | 89 | 9.6% |  | 146 | 3.5% | 84 | 5.5% |  |
| Others | 3 | 0.3% | 4 | 0.4% |  | 4 | 0.1% | 1 | 0.1% |  |
| Smoking status, n (%) |  |  |  |  | <0.001 |  |  |  |  | 0.001 |
| Never (<=100) | 311 | 26.2% | 182 | 19.7% |  | 3529 | 83.4% | 1331 | 87.3% |  |
| Past | 714 | 60.3% | 584 | 63.1% |  | 529 | 12.5% | 154 | 10.1% |  |
| Current(>=100) | 160 | 13.5% | 159 | 17.2% |  | 172 | 4.1% | 40 | 2.6% |  |
| Drinking status, n (%) |  |  |  |  | 0.354 |  |  |  |  | 0.003 |
| Never | 234 | 19.7% | 167 | 18.1% |  | 2330 | 55.1% | 907 | 59.5% |  |
| Past / Current | 951 | 80.3% | 758 | 81.9% |  | 1900 | 44.9% | 618 | 40.5% |  |
| DM, n (%) | 168 | 14.2% | 185 | 20.0% | <0.001 | 247 | 5.8% | 151 | 9.9% | <0.001 |
| HT, n (%) | 824 | 69.5% | 670 | 72.4% | 0.16 | 2146 | 50.7% | 895 | 58.7% | <0.001 |
| DL, n (%) | 211 | 17.8% | 158 | 17.1% | 0.706 | 842 | 19.9% | 322 | 21.1% | 0.332 |
| Number of teeth, n (%) |  |  |  |  | 0.001 |  |  |  |  | <0.001 |
| >= 20 | 977 | 82.4% | 704 | 76.1% |  | 3634 | 85.9% | 1214 | 79.6% |  |
| 10 to 19 | 121 | 10.2% | 133 | 14.4% |  | 447 | 10.6% | 221 | 14.5% |  |
| < 9 | 87 | 7.3% | 88 | 9.5% |  | 149 | 3.5% | 90 | 5.9% |  |
| Energy intake per day (kcal) | 1950.5 [1547.1, 2377.9] |  | 1992.8 [1605.5, 2446.9] |  | 0.03 | 1748.4 [1409.1, 2162] |  | 1760.1 [1422.9, 2200.6] |  | 0.302 |
| Seafood intake (g) | 54.3 [33.5, 83.3] |  | 58.5 [33.5, 85.4] |  | 0.149 | 58.8 [37.8, 87.6] |  | 60.7 [38.0, 91.5] |  | 0.115 |
| n-3 PUFA intake (g) | 2.3 [1.7, 2.7] |  | 2.2 [1.7, 2.8] |  | 0.627 | 2.5 [2.1, 3.0] |  | 2.5 [2.1, 3.0] |  | 0.373 |
| n-6 PUFA intake (g) | 11 [9.2, 12.9] |  | 10.9 [9.1, 12.8] |  | 0.492 | 12.3 [10.7, 14] |  | 12.2 [10.5, 14.0] |  | 0.325 |
| PUFA intake (g) | 13.4 [11.2, 15.6] |  | 13.2 [10.9, 15.6] |  | 0.584 | 15.0 [13.0, 16.9] |  | 14.9 [12.9, 17.0] |  | 0.607 |
| MUFA intake (g) | 23.9 [19.4, 28.2] |  | 23 [19, 27.4] |  | 0.026 | 27.2 [23.7, 30.9] |  | 26.7 [23.0, 30.6] |  | 0.008 |
| SFA intake (g) | 19.9 [16.1, 24.1] |  | 18.8 [15.3, 23.2] |  | <0.001 | 23.2 [19.9, 26.6] |  | 22.6 [19.2, 26.2] |  | <0.001 |
| TC (mg/dL) | 197 [175, 220] |  | 190 [170, 211] |  | <0.001 | 216 [195, 240] |  | 213 [192, 237] |  | <0.001 |
| HDL-C (mg/dL) | 56 [47, 68] |  | 54 [46, 65] |  | 0.016 | 69 [58, 81] |  | 66 [56, 78] |  | <0.001 |
| LDL-C (mg/dL) | 115 [95, 136] |  | 110 [90, 129] |  | <0.001 | 125 [105, 145] |  | 122 [102, 144] |  | 0.007 |
| TG (mg/dL) | 120 [81, 175] |  | 122 [85, 182] |  | 0.248 | 101 [72, 145] |  | 105 [75, 147] |  | 0.026 |

**Supplementary Table 3. Characteristics of participants according to hearing loss status by sex among those aged 65–79 years**

PTA, pure-tone average; dB HL, decibel hearing level; BMI, body mass index; DM, diabetes mellitus; HT, hypertension; DL, dyslipidemia; PUFA, polyunsaturated fatty acid; MUFA, monounsaturated fatty acid; SFA, saturated fatty acid; TC, total cholesterol; HDL-C, high-density lipoprotein cholesterol; LDL-C, low-density lipoprotein cholesterol; TG, triglycerides.

| 65-79 years | Men | | | | | Women | | | | |
| --- | --- | --- | --- | --- | --- | --- | --- | --- | --- | --- |
|  | Normal |  | Hearing loss |  | p-value | Normal |  | Hearing loss |  | p-value |
| Number | 877 |  | 1970 |  |  | 1328 |  | 1868 |  |  |
| PTA (dB HL) | 18.8 [16.2, 21.2] |  | 35 [30, 42.5] |  | <0.001 | 18.8 [15.0, 21.2] |  | 33.8 [28.8, 40.0] |  | <0.001 |
| Age (years) | 68 [66, 70] |  | 69 [66, 72] |  | <0.001 | 67 [66, 69] |  | 69 [66, 71] |  | <0.001 |
| BMI (kg/m²) | 23.4 [21.8, 25.1] |  | 23.5 [21.8, 25.5] |  | 0.236 | 22.4 [20.4, 24.4] |  | 22.6 [20.7, 25.1] |  | 0.004 |
| Family history of hearing loss, n (%) | 20 | 2.3% | 39 | 2.0% | 0.36 | 32 | 2.4% | 72 | 3.9% | 0.03 |
| Noise risk occupation, n (%) | 24 | 2.7% | 71 | 3.6% | 0.003 | 21 | 1.6% | 40 | 2.1% | 0.313 |
| History of cardiovascular disease, n (%) | 353 | 40.3% | 584 | 29.6% | 0.008 | 144 | 10.8% | 216 | 11.6% | 0.564 |
| Type of cohort, n (%) |  |  |  |  |  |  |  |  |  |  |
| Community-Based Cohort | 755 | 86.1% | 1822 | 92.5% | <0.001 | 1177 | 88.6% | 1738 | 93.0% | <0.001 |
| Birth and Three-Generation Cohort | 122 | 13.9% | 148 | 7.5% |  | 151 | 11.4% | 130 | 7.0% |  |
| Living with spouse, n (%) | 794 | 90.5% | 1767 | 89.7% | 0.609 | 884 | 66.6% | 1202 | 64.3% | 0.208 |
| Living with household members, n (%) | 833 | 95.0% | 200 | 10.2% | 0.008 | 1073 | 80.8% | 1493 | 79.9% | 0.571 |
| Educational status, n (%) |  |  |  |  | <0.001 |  |  |  |  | 0.001 |
| High (college and above) | 287 | 32.7% | 448 | 22.7% |  | 108 | 8.1% | 100 | 5.4% |  |
| Middle (high school) | 69 | 7.9% | 294 | 14.9% |  | 1106 | 83.3% | 1569 | 84.0% |  |
| Low (junior high school and below) | 517 | 59.0% | 1226 | 62.2% |  | 110 | 8.3% | 197 | 10.5% |  |
| Others | 4 | 0.5% | 2 | 0.1% |  | 4 | 0.3% | 2 | 0.1% |  |
| Smoking status, n (%) |  |  |  |  | 0.856 |  |  |  |  | 0.358 |
| Never (<=100) | 68 | 7.8% | 156 | 7.9% |  | 1243 | 93.6% | 1767 | 94.6% |  |
| Past | 544 | 62.0% | 1239 | 62.9% |  | 72 | 5.4% | 81 | 4.3% |  |
| Current (>=100) | 265 | 30.2% | 575 | 29.2% |  | 13 | 1.0% | 20 | 1.1% |  |
| Drinking status, n (%) |  |  |  |  | 0.998 |  |  |  |  | 0.535 |
| Never | 192 | 21.9% | 433 | 22.0% |  | 881 | 66.3% | 1260 | 67.5% |  |
| Past / Current | 685 | 78.1% | 1537 | 78.0% |  | 447 | 33.7% | 608 | 32.5% |  |
| DM, n (%) | 153 | 17.4% | 313 | 15.9% | 0.326 | 116 | 8.7% | 212 | 11.3% | 0.019 |
| HT, n (%) | 671 | 76.5% | 1461 | 74.2% | 0.198 | 869 | 65.4% | 1296 | 69.4% | 0.021 |
| DL, n (%) | 142 | 17.1% | 260 | 14.4% | 0.039 | 316 | 23.8% | 413 | 22.1% | 0.282 |
| Number of teeth, n (%) |  |  |  |  | <0.001 |  |  |  |  | 0.001 |
| >= 20 | 636 | 72.5% | 1253 | 63.6% |  | 945 | 71.2% | 1217 | 65.1% |  |
| 10 to 19 | 140 | 16.0% | 418 | 21.2% |  | 245 | 18.4% | 428 | 22.9% |  |
| < 9 | 101 | 11.5% | 299 | 15.2% |  | 138 | 10.4% | 223 | 11.9% |  |
| Energy intake per day (kcal) | 1986.5 [1594.8, 2435] |  | 2003.8 [1593, 2449.4] |  | 0.422 | 1899.2 [1540.8, 2392.8] |  | 1866.5 [1478.4, 2349] |  | 0.041 |
| Seafood intake (g) | 61.4 [36.8, 92.8] |  | 60.2 [35.7, 92.1] |  | 0.675 | 63.2 [39.2, 97.0] |  | 66.1 [40.6, 104.6] |  | 0.079 |
| n-3 PUFA intake (g) | 2.4 [1.9, 2.9] |  | 2.4 [1.9, 2.9] |  | 0.726 | 2.6 [2.1, 3.1] |  | 2.6 [2.1, 3.1] |  | 0.962 |
| n-6 PUFA intake (g) | 11.6 [9.7, 13.5] |  | 11.3 [9.5, 13.3] |  | 0.089 | 12.1 [10.4, 14.0] |  | 12.2 [10.5, 13.9] |  | 0.803 |
| PUFA intake (g) | 14.1 [11.9, 16.4] |  | 13.9 [11.6, 16.2] |  | 0.174 | 14.8 [12.7, 17.1] |  | 14.9 [12.8, 17.1] |  | 0.872 |
| MUFA intake (g) | 25.1 [20.7, 29.7] |  | 24.4 [20.3, 28.9] |  | 0.034 | 27.5 [23.7, 31.5] |  | 27.4 [23.6, 31.2] |  | 0.761 |
| SFA intake (g) | 21.4 [17.9, 25] |  | 20.6 [16.9, 24.4] |  | <0.001 | 22.8 [19.3, 26.7] |  | 23.1 [19.6, 26.5] |  | 0.686 |
| TC (mg/dL) | 190 [169, 211] |  | 188 [166, 208] |  | 0.06 | 210 [188, 231] |  | 207 [185, 230] |  | 0.062 |
| HDL-C (mg/dL) | 57 [48, 68] |  | 56 [47, 67] |  | 0.072 | 68 [57, 79] |  | 66 [56, 77] |  | 0.008 |
| LDL-C (mg/dL) | 109 [90, 127] |  | 107 [88, 125] |  | 0.168 | 118 [101, 139] |  | 117 [99, 137] |  | 0.114 |
| TG (mg/dL) | 108 [77, 159] |  | 109 [76, 159] |  | 0.675 | 103 [74, 143] |  | 107 [77, 149] |  | 0.039 |

**Supplementary Table 4. Multivariable-adjusted β coefficients for hearing thresholds (dB HL) according to quartiles of SFA intake in men aged 50–64 and 65–79 years**

PTA, pure-tone average; Q1, lowest quartile; Q2, second quartile; Q3, third quartile; Q4, highest quartile; CI, confidence interval; SFA, saturated fatty acid; dB HL, decibel hearing level.

| SFA | Men (50-64 years, n=2110) | | | | | SFA | Men (65-79 years, n=2847) | | | | |
| --- | --- | --- | --- | --- | --- | --- | --- | --- | --- | --- | --- |
| PTA | β coefficient | 95% CI: lower | 95% CI: upper | p-value | FDR-adjusted P value | PTA | β coefficient | 95% CI: lower | 95% CI: upper | p-value | FDR-adjusted P value |
| Q1 (< 15.7g) | Reference | — | — | — | — | Q1 (< 17.2 g) | Reference | — | — | — | — |
| Q2 (15.7–19.5 g) | -1.017 | -2.312 | 0.279 | 0.124 | 0.325 | Q2 (17.2–20.9 g) | -0.324 | -1.623 | 0.975 | 0.625 | 0.914 |
| Q3 (19.5–23.6 g) | -1.836 | -3.28 | -0.392 | 0.013 | 0.199 | Q3 (20.9–24.6 g) | -1.062 | -2.491 | 0.368 | 0.146 | 0.914 |
| Q4 ( >= 23.6 g) | -1.86 | -3.505 | -0.215 | 0.027 | 0.199 | Q4 ( >= 24.6 g) | -1.768 | -3.399 | -0.137 | 0.034 | 0.353 |
| 500 Hz | β coefficient | 95% CI: lower | 95% CI: upper | p-value | FDR-adjusted P value | 500 Hz | β coefficient | 95% CI: lower | 95% CI: upper | p-value | FDR-adjusted P value |
| Q1 (< 15.7g) | Reference | — | — | — | — | Q1 (< 17.2 g) | Reference | — | — | — | — |
| Q2 (15.7–19.5 g) | -0.27 | -1.508 | 0.967 | 0.668 | 0.908 | Q2 (17.2–20.9 g) | -0.129 | -1.406 | 1.148 | 0.843 | 0.981 |
| Q3 (19.5–23.6 g) | -1.662 | -3.042 | -0.283 | 0.018 | 0.347 | Q3 (20.9–24.6 g) | 0.232 | -1.173 | 1.638 | 0.746 | 0.981 |
| Q4 ( >= 23.6 g) | -1.574 | -3.145 | -0.003 | 0.05 | 0.347 | Q4 ( >= 24.6 g) | -0.656 | -2.26 | 0.947 | 0.422 | 0.981 |
| 1000 Hz | β coefficient | 95% CI: lower | 95% CI: upper | p-value | FDR-adjusted P value | 1000 Hz | β coefficient | 95% CI: lower | 95% CI: upper | p-value | FDR-adjusted P value |
| Q1 (< 15.7g) | Reference | — | — | — | — | Q1 (< 17.2 g) | Reference | — | — | — | — |
| Q2 (15.7–19.5 g) | -1.181 | -2.5 | 0.137 | 0.079 | 0.415 | Q2 (17.2–20.9 g) | -1.298 | -2.65 | 0.054 | 0.06 | 0.579 |
| Q3 (19.5–23.6 g) | -1.805 | -3.275 | -0.336 | 0.016 | 0.169 | Q3 (20.9–24.6 g) | -1.317 | -2.805 | 0.171 | 0.083 | 0.579 |
| Q4 ( >= 23.6 g) | -2.081 | -3.755 | -0.407 | 0.015 | 0.169 | Q4 ( >= 24.6 g) | -2.564 | -4.262 | -0.866 | 0.003 | 0.065 |
| 2000 Hz | β coefficient | 95% CI: lower | 95% CI: upper | p-value | FDR-adjusted P value | 2000 Hz | β coefficient | 95% CI: lower | 95% CI: upper | p-value | FDR-adjusted P value |
| Q1 (< 15.7g) | Reference | — | — | — | — | Q1 (< 17.2 g) | Reference | — | — | — | — |
| Q2 (15.7–19.5 g) | -0.889 | -2.513 | 0.734 | 0.283 | 0.606 | Q2 (17.2–20.9 g) | -0.537 | -2.169 | 1.095 | 0.519 | 0.838 |
| Q3 (19.5–23.6 g) | -1.489 | -3.3 | 0.321 | 0.107 | 0.32 | Q3 (20.9–24.6 g) | -1.456 | -3.252 | 0.34 | 0.112 | 0.707 |
| Q4 ( >= 23.6 g) | -1.292 | -3.355 | 0.77 | 0.219 | 0.575 | Q4 ( >= 24.6 g) | -1.887 | -3.937 | 0.163 | 0.071 | 0.707 |
| 4000 Hz | β coefficient | 95% CI: lower | 95% CI: upper | p-value | FDR-adjusted P value | 4000 Hz | β coefficient | 95% CI: lower | 95% CI: upper | p-value | FDR-adjusted P value |
| Q1 (< 15.7g) | Reference | — | — | — | — | Q1 (< 17.2 g) | Reference | — | — | — | — |
| Q2 (15.7–19.5 g) | -1.947 | -4.12 | 0.226 | 0.079 | 0.277 | Q2 (17.2–20.9 g) | 0.826 | -1.092 | 2.745 | 0.398 | 0.757 |
| Q3 (19.5–23.6 g) | -2.337 | -4.76 | 0.086 | 0.059 | 0.26 | Q3 (20.9–24.6 g) | -1.673 | -3.785 | 0.438 | 0.12 | 0.757 |
| Q4 ( >= 23.6 g) | -2.072 | -4.832 | 0.688 | 0.141 | 0.328 | Q4 ( >= 24.6 g) | -2.36 | -4.769 | 0.049 | 0.055 | 0.576 |

**Supplementary Table 5. Multivariable-adjusted β coefficients for hearing thresholds (dB HL) according to quartiles of SFA intake in women aged 50–64 and 65–79 years**

PTA, pure-tone average; Q1, lowest quartile; Q2, second quartile; Q3, third quartile; Q4, highest quartile; CI, confidence interval; SFA, saturated fatty acid; dB HL, decibel hearing level.

| SFA | Women (50-64 years, n=5755) | | | | | SFA | Women (65-79 years, n=3196) | | | | |
| --- | --- | --- | --- | --- | --- | --- | --- | --- | --- | --- | --- |
| PTA | β coefficient | 95% CI: lower | 95% CI: upper | p-value | FDR-adjusted P value | PTA | β coefficient | 95% CI: lower | 95% CI: upper | p-value | FDR-adjusted P value |
| Q1 (< 19.6g) | Reference | — | — | — | — | Q1 (< 19.5 g) | Reference | — | — | — | — |
| Q2 (19.6–23.0 g) | -0.213 | -0.873 | 0.446 | 0.527 | 0.868 | Q2 (19.5–23.0 g) | -0.757 | -1.863 | 0.349 | 0.18 | 0.791 |
| Q3 (23.0–26.5 g) | -0.361 | -1.051 | 0.329 | 0.305 | 0.868 | Q3 (23.0–26.6 g) | 0.743 | -0.405 | 1.89 | 0.204 | 0.791 |
| Q4 ( >= 26.5 g) | -0.242 | -0.967 | 0.483 | 0.513 | 0.868 | Q4 ( >= 26.6 g) | -0.422 | -1.631 | 0.787 | 0.494 | 0.812 |
| 500 Hz | β coefficient | 95% CI: lower | 95% CI: upper | p-value | FDR-adjusted P value | 500 Hz | β coefficient | 95% CI: lower | 95% CI: upper | p-value | FDR-adjusted P value |
| Q1 (< 19.6g) | Reference | — | — | — | — | Q1 (< 19.5 g) | Reference | — | — | — | — |
| Q2 (19.6–23.0 g) | 0.174 | -0.506 | 0.854 | 0.616 | 0.79 | Q2 (19.5–23.0 g) | -0.641 | -1.788 | 0.506 | 0.273 | 0.818 |
| Q3 (23.0–26.5 g) | -0.378 | -1.089 | 0.333 | 0.297 | 0.727 | Q3 (23.0–26.6 g) | 1.313 | 0.123 | 2.502 | 0.031 | 0.545 |
| Q4 ( >= 26.5 g) | -0.032 | -0.779 | 0.715 | 0.933 | 0.98 | Q4 ( >= 26.6 g) | 0.043 | -1.211 | 1.297 | 0.946 | 0.994 |
| 1000 Hz | β coefficient | 95% CI: lower | 95% CI: upper | p-value | FDR-adjusted P value | 1000 Hz | β coefficient | 95% CI: lower | 95% CI: upper | p-value | FDR-adjusted P value |
| Q1 (< 19.6g) | Reference | — | — | — | — | Q1 (< 19.5 g) | Reference | — | — | — | — |
| Q2 (19.6–23.0 g) | -0.502 | -1.221 | 0.217 | 0.171 | 0.599 | Q2 (19.5–23.0 g) | -0.795 | -1.989 | 0.398 | 0.192 | 0.76 |
| Q3 (23.0–26.5 g) | -0.843 | -1.595 | -0.091 | 0.028 | 0.294 | Q3 (23.0–26.6 g) | 0.361 | -0.877 | 1.599 | 0.568 | 0.873 |
| Q4 ( >= 26.5 g) | -0.65 | -1.44 | 0.141 | 0.107 | 0.45 | Q4 ( >= 26.6 g) | -0.314 | -1.619 | 0.991 | 0.637 | 0.873 |
| 2000 Hz | β coefficient | 95% CI: lower | 95% CI: upper | p-value | FDR-adjusted P value | 2000 Hz | β coefficient | 95% CI: lower | 95% CI: upper | p-value | FDR-adjusted P value |
| Q1 (< 19.6g) | Reference | — | — | — | — | Q1 (< 19.5 g) | Reference | — | — | — | — |
| Q2 (19.6–23.0 g) | -0.231 | -1.044 | 0.581 | 0.577 | 0.988 | Q2 (19.5–23.0 g) | -0.688 | -1.997 | 0.621 | 0.303 | 0.587 |
| Q3 (23.0–26.5 g) | -0.058 | -0.907 | 0.792 | 0.894 | 0.988 | Q3 (23.0–26.6 g) | 0.857 | -0.501 | 2.215 | 0.216 | 0.587 |
| Q4 ( >= 26.5 g) | -0.039 | -0.933 | 0.854 | 0.931 | 0.988 | Q4 ( >= 26.6 g) | -0.399 | -1.831 | 1.032 | 0.584 | 0.876 |
| 4000 Hz | β coefficient | 95% CI: lower | 95% CI: upper | p-value | FDR-adjusted P value | 4000 Hz | β coefficient | 95% CI: lower | 95% CI: upper | p-value | FDR-adjusted P value |
| Q1 (< 19.6g) | Reference | — | — | — | — | Q1 (< 19.5 g) | Reference | — | — | — | — |
| Q2 (19.6–23.0 g) | -0.274 | -1.187 | 0.64 | 0.557 | 0.905 | Q2 (19.5–23.0 g) | -1.309 | -2.828 | 0.21 | 0.091 | 0.827 |
| Q3 (23.0–26.5 g) | -0.07 | -1.025 | 0.886 | 0.887 | 0.962 | Q3 (23.0–26.6 g) | 0.258 | -1.318 | 1.834 | 0.748 | 0.827 |
| Q4 ( >= 26.5 g) | -0.023 | -1.028 | 0.982 | 0.964 | 0.964 | Q4 ( >= 26.6 g) | -1.059 | -2.721 | 0.602 | 0.211 | 0.827 |
